# Molecular Dynamics Simulation on Mechanical and Piezoelectric Properties of Boron Nitride Honeycomb Structures

**DOI:** 10.3390/nano9071044

**Published:** 2019-07-21

**Authors:** Lu Xie, Tianhua Wang, Chenwei He, Zhihui Sun, Qing Peng

**Affiliations:** 1 School of Mechanical Engineering, University of Science and Technology Beijing, Beijing 100083, China; 2 Reactor Engineering and Safety Research Center, China Nuclear Power Technology Research Institute Co., Ltd., Shenzhen 518031, China; 3 Nuclear Engineering and Radiological Sciences, University of Michigan, Ann Arbor, MI 48108, USA

**Keywords:** boron nitride honeycomb, molecular dynamics simulation, mechanical property, piezoelectric property

## Abstract

Boron nitride honeycomb structure is a new three-dimensional material similar to carbon honeycomb, which has attracted a great deal of attention due to its special structure and properties. In this paper, the tensile mechanical properties of boron nitride honeycomb structures in the zigzag, armchair and axial directions are studied at room temperature by using molecular dynamics simulations. Effects of temperature and strain rate on mechanical properties are also discussed. According to the observed tensile mechanical properties, the piezoelectric effect in the zigzag direction was analyzed for boron nitride honeycomb structures. The obtained results showed that the failure strains of boron nitride honeycomb structures under tensile loading were up to 0.83, 0.78 and 0.55 in the armchair, zigzag and axial directions, respectively, at room temperature. These findings indicated that boron nitride honeycomb structures have excellent ductility at room temperature. Moreover, temperature had a significant effect on the mechanical and tensile mechanical properties of boron nitride honeycomb structures, which can be improved by lowering the temperature within a certain range. In addition, strain rate affected the maximum tensile strength and failure strain of boron nitride honeycomb structures. Furthermore, due to the unique polarization of boron nitride honeycomb structures, they possessed an excellent piezoelectric effect. The piezoelectric coefficient e obtained from molecular dynamics was $0.702\;{C/m}^{2}$, which was lower than that of the monolayer boron nitride honeycomb structures, $e=0.79^{\;}{C/m}^{2}$. Such excellent piezoelectric properties and failure strain detected in boron nitride honeycomb structures suggest a broad prospect for the application of these new materials in novel nanodevices with ultrahigh tensile mechanical properties and ultralight-weight materials.

## 1. Introduction

Boron nitride (BN) has a similar structure to graphene and exhibits excellent mechanical and electrical properties [1,2,3,4,5]. The two-dimensional BN films have been successfully stripped out by using micromechanical cleavage. These structures reveal high crystal quality and macroscopic continuity [6]. BN nanobelts are fabricated by a simple ZnS nanoribbon templating method and possess good optical properties [7]. The wide application of two-dimensional materials in various fields has attracted the interest of different research groups in three-dimensional materials. A novel boron nitride honeycomb (BNHC) structure consisting of zigzag-edged BN nanosheets is proposed by Wu et al. [8] and they confirmed structural stability of this material. In particular, carbon honeycomb (CHC) structures which are similar to BNHCs, have been successfully fabricated [9]. These honeycomb structures can be used not only for storing various gases and liquids but also as a matrix for new composite materials.

Since the first report by Wang et al. on a prototype nanogenerator based on zinc oxide nanowires [10], piezoelectric nanomaterials have received extensive attention [11,12,13]. It has been found that BN belongs to the piezoelectric materials [14] and shows good piezoelectric effect [4]. The piezoelectric effect means that when an external pressure is applied to a piezoelectric material, a potential difference is generated on the surface of the material. Conversely, a piezoelectric material deforms when an external electric field is applied to it. The essence is that when pressure is exploited to a piezoelectric material, the non-centrosymmetric ions inside the crystal starts to be polarized and results in a potential difference. Owing to the simultaneous possession of piezoelectricity and semiconductor properties, the piezopotential created in the crystal has a strong effect on the carrier transport at the interface/junction. The piezoelectric potential produced by the mechanical deformation of a piezoelectric material can be used as the gate voltage to change the carrier transmission characteristics [15] and thereby improves the performance of photovoltaic devices such as nanosensors [16], nanogenerators [17], nanotransistors [18] and so forth. According to the density functional theory (DFT) calculations, it is found that BN nanosheets exhibit stronger piezoelectric coupling than conventional bulky Wurtzite structures [4]. The piezoelectric effect of BNHC structures has been analyzed by using a combination of finite element and molecular dynamics simulations and studies have indicated that BNHC structures provide a good piezoelectric effect and piezopotential properties which can be efficiently adjusted by regulating the lattice constant [19].

Since mechanical properties of a material directly affect its application in various fields, therefore, it is necessary to study this important parameter. There are many studies on the mechanical properties of BN nanotubes, for example, the elastic properties of an individual multi-wall BN nanotube is determined experimentally and the results confirm that these nanotubes are highly crystalline with few defects [20]. Moreover, mechanical properties of monolayer systems of honeycomb structures are investigated by using an equation of state (EOS). The results indicate that graphene is the most elastic, followed by BN films and both materials have considerable strength [21]. Inspired by the excellent mechanical properties and wide applications of 2D materials, such as BN nanosheets and graphene, it is reasonable to build three-dimensional materials with excellent mechanical properties. Some studies have shown that mechanical properties of CHC structures bear a strong cell-size effect and anisotropy. The failure stress and failure strain in different directions decrease with enhancing the cell-size [22]. However, for BNHC structures, scholars focus on their electrical properties instead of mechanical properties. The mechanical properties of BNHC structures are the premise to ensure other properties. Therefore, mechanical properties of BNHC structures require further attentions. There are also relatively few analyses on the mechanical properties of BNHC structures using molecular dynamics (MD) simulations.

Motivated by these ideas, a boron nitride honeycomb structure was constructed based on the BN nanosheets in this paper. The structures possessed good mechanical properties and piezoelectric effect and tensile mechanical properties of the structures in different directions were studied by using MD simulations. The effects of temperature and strain rate on tensile mechanical properties were also analyzed. Based on the above studies, the piezoelectric properties of BNHCs with a specific lattice constant in the zigzag direction were further affirmed.

## 2. Materials and Methods

**Figure 1:**
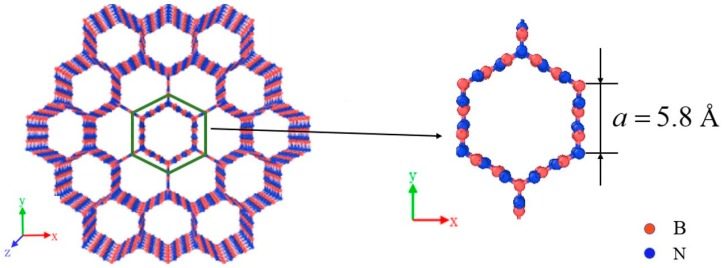
The graphical structure of boron nitride honeycomb.

In this research, a new three-dimensional boron nitride honeycomb structure is established as shown in Figure 1. It can be viewed as consisting of zigzag-edged BN nanoribbons with sp^2^ bonding in the wall and sp^3^ bonding in junction. The cross-section perpendicular to the axial (cell axis) is a honeycomb structure based on regular hexagon with a side length 5.8 Å.

Based on the established model, the tensile mechanical properties and piezoelectric behaviors were studied by MD simulations. Herein, all MD simulations were conducted using the large-scale atomic/molecular massively parallel simulator (LAMMPS) [23]. The structural analysis and data processing of BNHCs were conducted by using the visualization software OVITO [24]. In all the simulations, the interactions between B and N atoms were described by Tersoff potential [25]. In the present simulations, the values of parameters in the Tersoff potential were taken from Reference [26]. This Tersoff potential has been successfully employed to evaluate the mechanical and piezoelectric properties of BN [27,28]. We tested three potentials of Tersoff, extep and Reaxff for BNHCs and found that the Tersoff potential is the most suitable (see the Supplementary Files for details). The curves of the total energy and temperature of BNHCs were obtained (see Figure S1 in the Supplementary Files). Time step is 0.5 fs. The isothermal-isobaric (NPT) ensemble was utilized to update the positions and velocities of the atoms by using the Nosé-Hoover temperature thermostat [29]. In addition, the velocity Verlet algorithm was employed to integrate the Hamiltonian equations of the determined motion. Before analysis of performance of the model, the conjugate gradient algorithm was used to calculate the energy minimization of BNHCs to obtain their stable states. Then, BNHCs were relaxed at room temperature (300 K) for 25 ps to obtain a state of equilibrium. After relaxation, the model was contained to a total of 2016 atoms and size of the model was 30.32 Å × 35.01 Å × 29.52 Å.

### 2.1. MD Simulations for Tensile Mechanical Properties of BNHCs

First, the equilibrium system was gradually heated from 300 to 5000 K at a heating rate of $4.7\times 10^{12}\;K\cdot s^{-1}$ and the melting characteristics of BNHCs were analyzed. After the system was fully relaxed, the mechanical properties of BNHCs were analyzed. In this paper, the tensile mechanical properties of BNHCs in the zigzag (*x*-axis), armchair (*y*-axis) and axial (*z*-axis) directions were studied. Thereafter, the influences of temperature and strain rate were analyzed on the mechanical properties of BNHCs. The strain load was applied in different directions and the Young’s modulus and the maximum tensile strength were obtained according to the stress-strain curves. The engineering strain rate was taken as $10^{9}\;s^{-1}$ in the simulation.

### 2.2. MD Simulations for Piezoelectric Properties of BNHCs

According to the studies of mechanical properties, the piezoelectric behaviors of the zigzag direction of BNHCs were investigated. Piezoelectric effect refers to the influence of external pressure on a piezoelectric material, in which the material produces a potential difference on its surface and causes deformation of the material due to the generation of an external electric field. Therefore, the piezoelectric effect is essentially dependent on the deformation of the material due to external forces, which polarizes the internal material. The piezoelectric constant is a parameter that characterizes the polarization of a piezoelectric material under an external pressure. Herein, the MD simulations were used to apply the strain load to the zigzag direction of BNHCs. The piezoelectric constant was obtained by calculating the polarization inside BNHCs and, then, piezoelectric characteristics were evaluated.

At a temperature of 300 K, a strain load was applied to the zigzag direction of BNHCs and the polarization of *P* and strain of *ε* were recorded in the zigzag direction. The piezoelectric constant of *e* was obtained by calculating the slopes of the polarization (*P*) and strain (*ε*) [30].

## 3. Results and Discussion

**Figure 2:**
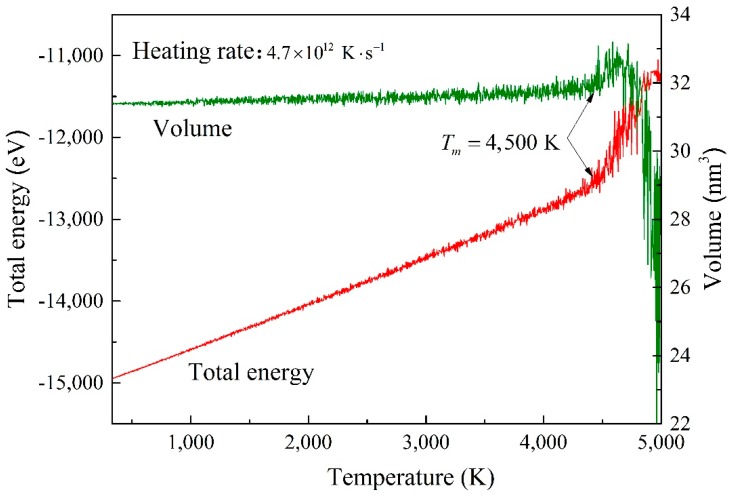
The curves of total energy and volume versus temperature during heating of boron nitride honeycombs (BNHCs).

Before analysis of the mechanical properties, the system was warmed-up. The curve of the total energy, volume and temperature of BNHCs were obtained at a heating rate of $4.7\times 10^{12}\;K\cdot s^{-1}$, as shown in Figure 2.

Figure 2 shows that the total energy and volume of BNHCs increased linearly with increasing temperature during the heating process. However, when the temperature reached about 4500 K, the total energy inclined significantly, and the maximum volume was also reached. When the temperature continued to increase, the system-energy was also enhanced and the volume dropped sharply. This finding indicated that the phase of BNHC structure was changed at about 4500 K and it began to melt. The obtained melting point of BNHCs by MD simulations was around 4500 K. It is found that CHCs maintains structural stability at 2000 K [31], which confirmed the high thermal stability of BNHCs.

### 3.1. Tensile Mechanical Properties of BNHCs

**Figure 3:**
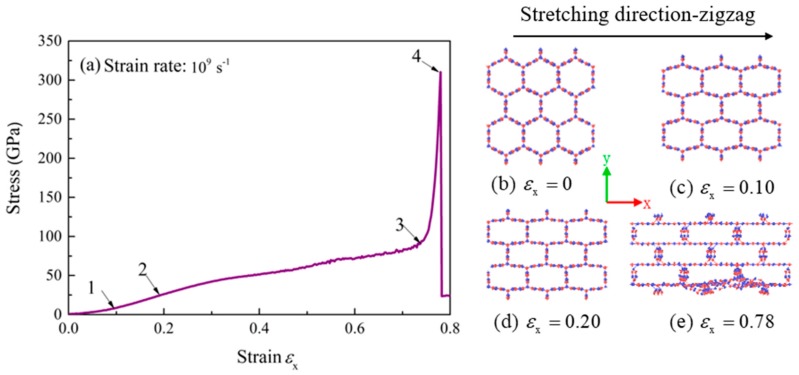
(**a**) The stress-strain curve of BNHCs under tensile loading along the zigzag direction; (**b**)–(**e**) Deformation of BNHCs under different tensile strains along the zigzag direction.

In this section, the mechanical properties of BNHCs under tensile loading along the zigzag (*x*-axis), armchair (*y*-axis) and axial (*z*-axis) directions were studied at room temperature (300 K). At a strain rate of $10^{9}{\;s}^{-1}$, the stress-strain curve along the zigzag direction is shown in Figure 3.

It can be seen from the stress-strain curve in Figure 3a that the change of the bond angle played a leading role in the initial stage of stretching. At the stretching of 0.1 (point 1), the bond angle and B-N bond length together caused the fast increase of stress. When the stretching was continued until the strain of 0.2 (point 2), the rate of stress was slowed down, because the increase of strain had been due to the elongation of B-N bond. The tensile strength of BNHCs in the zigzag direction did not show a good yielding stage and the strengthening phase began from the strain of 0.76 (point 3). When the strain was increased to 0.78 (point 4), the stress was lowered sharply, and the material failed. The Young’s modulus of BNHCs under tensile loading along the zigzag direction was calculated as about 146 GPa and the maximum tensile strength of 310 GPa was observed at the strain of 0.78.

Figure 3b–e show the tensile deformation process of BNHCs in the zigzag direction. As these figures show, BNHCs had a regular hexagonal structure when unstretched and this structure was basically maintained before the strain of 0.1. This finding revealed that the stress was increased linearly with strain; when the strain reached 0.2, the cross section of BNHCs was transformed into a rectangular structure until failure occurred at the strain of 0.78. This perception structurally demonstrated that elongation of the bond length caused an increase in the strain during the above stretching phase.

**Figure 4:**
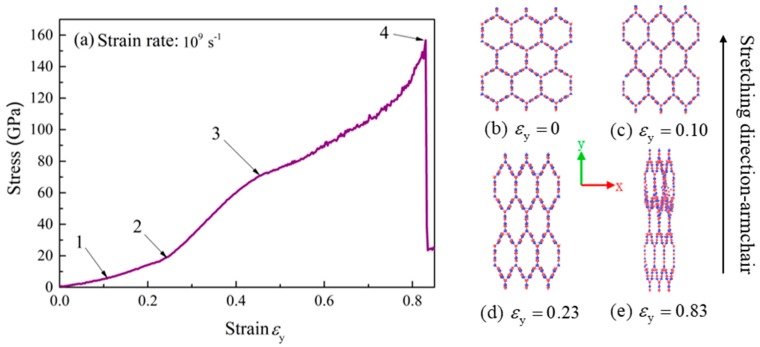
(**a**) The stress-strain curve of BNHCs under tensile loading along the armchair direction; (**b**–**e**) Deformation of BNHCs under different tensile strains along the armchair direction.

The tensile stress-strain curve of BNHCs along the armchair is shown in Figure 4a. There are four stages in the stretching process: At the beginning of stretching, the stress was increased almost linearly with strain and increasing of strain during this process was mainly due to the change of B-N bond angle. When the strain reached 0.1 (point 1), the rate of stress-enhancement was increased because the bond length and bond angle are changing. When the strain approached 0.23 (point 2), the maximum rate of stress was attained, mainly due to the significant changes of B-N bond length. When the strain continued to increase up to 0.5 (point 3), only the bond length was altered, and the rate of stress was decreased. The maximum tensile strength of 156 GPa was achieved as the strain reached 0.83 (point 4). The strain increases and the structure eventually failed. The Young’s modulus of about 173 GPa was calculated for BNHCs under tensile loading along the armchair direction.

Figure 4b–e describe the deformation process of BNHCs as it is stretched along the armchair direction. It can be seen that when the stretching to strain was 0.1, the regular hexagonal cross section of BNHCs started to change due to alteration of the bond angle. When the strain was 0.23, the BNHCs completely deviated from the regular hexagon. Eventually, the structure was destroyed, and the material failed when the strain increased to 0.83.

**Figure 5:**
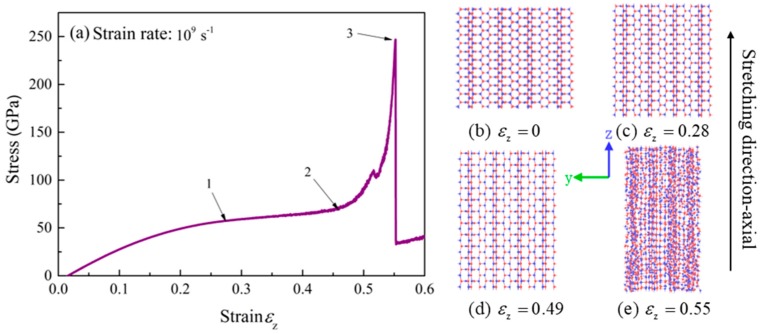
(**a**) The stress-strain curve of BNHCs under tensile loading along the axial direction; (**b**–**e**) Deformation of BNHCs under different tensile strains along the axial direction.

The stress-strain curve of BNHCs under tensile loading along the axial direction is shown in Figure 5a. The four stages of the stretching process were included (1) approximately linear increase of the stress with strain at the beginning of stretching, similar to the linear phase. (2) The strain showed a small change with strain at the stage of 0.28–0.49, which was approximated as the yield stage. The stresses were about 57 and 74 GPa at points 1 and 2, respectively. (3) When the strain was in the stage of 0.49–0.55, the deformation of the material was enhanced as the strain was increased, the strengthening stage appeared and the maximum tensile strength of 247 GPa was attained at point 3. (4) Finally, the structure was failed by continuation of stretch. The Young’s modulus of BNHCs under tensile loading along the axial direction was calculated as about 334 GPa.

Figure 5b–e describes the tensile deformation process for BNHCs along the axial direction. It was found that the axial direction grew in size as the strain increased, until a strain of 0.55, in which the structure was destroyed, and the material failed.

Table 1 lists the Young’s modulus, failure strain and maximum tensile strength of BNHCs under tensile in three different directions at room temperature. It can be envisaged that the largest Young’s modulus was obtained in the axial direction, whereas no significantly different values were gained for the zigzag and armchair directions. The axial direction showed the smallest failure strain, indicating that ductility in the axial direction was not good and ductility of the armchair direction was slightly higher than that of the zigzag direction. The maximum tensile strength was observed in the zigzag direction. The tensile processes and the evolution of shear stains of BNHCs in the zigzag, armchair and cell axis directions are presented, respectively (see Video S1–S3 in the Supplementary Files).

The failure strain of 0.76–0.81 was calculated for BNHCs along the armchair direction by using the combination of finite element method and molecular dynamics simulations [19]. The results of this investigation were consistent with the previous reports. The failure strains of CHC in the zigzag, armchair and cell axis directions were 0.204, 0.320 and 0.225, respectively. Moreover, the maximum tensile strengths of CHC in the zigzag, armchair and cell axis directions were 23.7, 22.4 and 55.3 GPa, respectively [32]. The failure strain of BNHCs reached about 0.8, which was significantly higher than that of CHC; the maximum tensile strength was also significantly higher than that of CHC, Compared to CHC, BNHCs showed high failure strain and improved mechanical properties. In addition, the failure strain of BNHCs was significantly higher than that of other piezoelectric materials. The failure strains of MoS_2_ nanosheets have been about 0.09 and 0.12 in the zigzag and armchair directions [33], whereas the failure strains of about 0.2 and 0.15 are obtained for GaN [34] and ZnO [35] nanowires, respectively.

### 3.2. Effect of Temperature on the Tensile Mechanical Properties of BNHCs

In practical engineering, the mechanical properties of materials often determine their applications. Many factors such as composition, stress state, nature of the load, environmental conditions and temperature affect properties of the material. In this section, effects of temperature and strain rate on the mechanical properties of BNHCs are mainly studied by using MD simulations.

**Figure 6:**
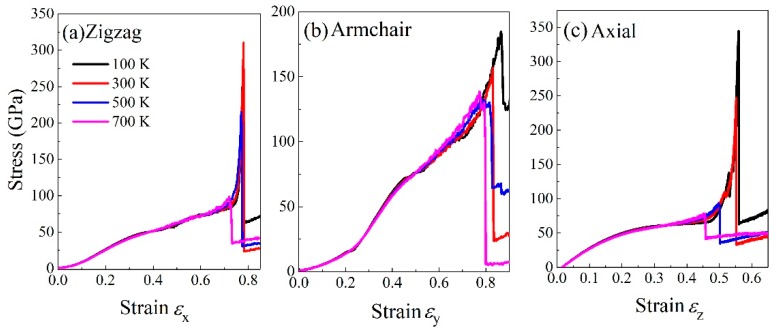
The stress-strain curves of BNHCs under tensile loading at various temperatures along (**a**) zigzag, (**b**) armchair and (**c**) axial directions, respectively.

Figure 6 shows the stress-strain curves of BNHCs under tensile loading in three different directions over a temperature range of 100–700 K. According to Figure 6a, when the structure was stretched in the zigzag direction, the maximum tensile strength of 310 GPa was attained at room temperature (300 K); but, this parameter was reduced 31.6% and reached to 98 GPa as the temperature raised-up to 700 K. Accordingly, the maximum tensile strength of 249 GPa was achieved at 100 K, which is 80.3% of that at room temperature. From the failure strain points of view, the failure strains of 0.775, 0.78, 0.772 and 0.73 were obtained at 100, 300, 500 and 700 K, respectively. Based on the above results, the maximum tensile strength and failure strain of BNHCs were achieved in the zigzag direction at room temperature, indicating the best strength at room temperature. However, the maximum tensile strength and failure strain were higher at low temperature. Therefore, the best properties of BNHCs in the zigzag direction were observed at low temperatures in the range of 100–700 K and the best tensile property was attained at room temperature.

It can be seen from Figure 6b that a good temperature dependence was gained when the BNHCs was stretched along the armchair direction. The maximum tensile strength and failure strain were decreased with enhancing temperature. The maximum tensile strength ranged from 120 to 184 GPa and the failure strain changed from 0.79 to 0.87, indicating that the excellent ductility of BNHCs was perceived in the armchair direction. The maximum tensile strength did not fluctuate significantly at different temperatures and the failure strain was greater than that in the zigzag direction.

As Figure 6c shows, the maximum tensile strength and failure strain in the cell-axis direction were lowered as the temperature grown up during stretching along the axial direction. The maximum range of tensile strength was78–341 GPa and the failure strain was 0.46–0.55. As the temperature was increased, the maximum tensile strength fluctuated greatly. The maximum tensile strength at 700 K was decreased 31.5% at room temperature. However, when the temperature was 100 K, the maximum tensile strength was 1.38 times of that at room temperature. This finding showed that similar to the zigzag direction, the strength was significantly higher at low temperatures.

### 3.3. Effect of Strain Rate on the Tensile Mechanical Properties of BNHCs

**Figure 7:**
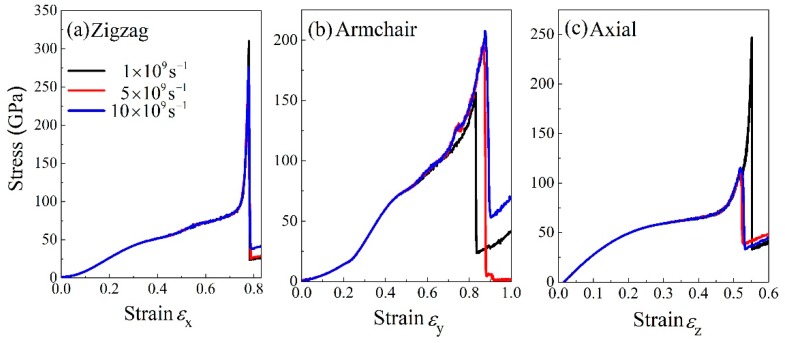
The stress-strain curves of BNHCs under tensile loading at various stain rates along (**a**) zigzag, (**b**) armchair and (**c**) axial directions, respectively.

To some extent, the strain rate also affects the mechanical properties of the material. Figure 7 show tensile stress-strain curves at three different strain rates. The strain rate slightly affected the tensile properties but did not show an obvious regularity. The stress-strain curve was unchanged in the initial stage of stretching and the strain rate only influenced the maximum tensile strength and failure strain. In the zigzag direction (Figure 7a), the maximum tensile strength was first decreased and then grew up as the strain rate was enhanced. In the armchair direction, as the strain rate was developed, the maximum tensile strength and failure strain rate were increased. In the axial direction, it reached to 247 GPa at a strain rate of $10^{9}\;s^{-1}$ and as the strain rate was inclined, the maximum tensile strength was decreased to 115 GPa. In short, the strain rate mainly affected the maximum tensile strength and failure strain of BNHCs. The stress-strain curve almost coincided in the initial stage of stretching. Due to different strain rates, the material broken up at different moments and resulted in various maximum tensile strengths.

### 3.4. Piezoelectric Properties of BNHCs

**Figure 8:**
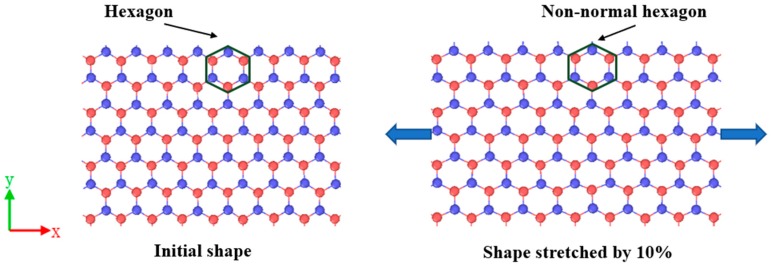
Atomic charge distribution of BN nanosheet under tensile loading.

In order to study the piezoelectric properties of BNHCs, first the strain load was applied to the monolayer of BNHCs along the zigzag direction. It was found that center of the triangle composed of three B atomic charges inside the structure did not coincide with the center of the triangle composed of three N atomic charges and the electric dipole moment caused polarization of the overall structure. Figure 8 shows a diagram for the distribution of atomic charge structures when BN nanosheet is stretched.

**Figure 9:**
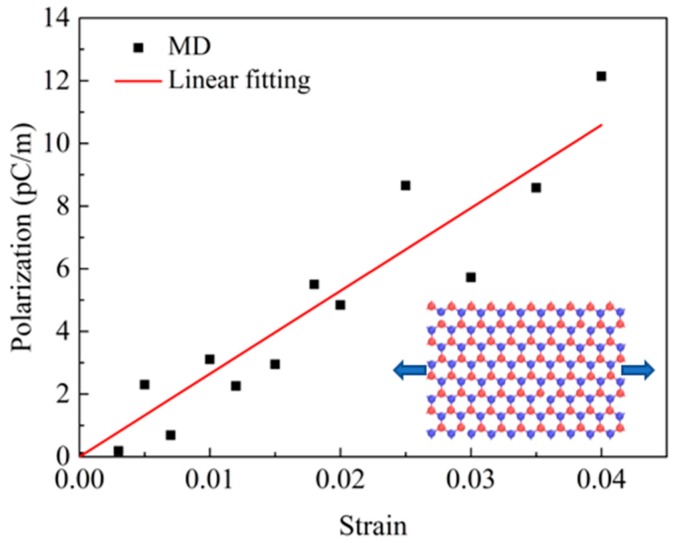
The polarization-strain curves of BN nanosheet under tensile loading along the zigzag direction obtained from molecular dynamics (MD) simulations.

The piezoelectric constant is a parameter that characterizes the internal polarization of a piezoelectric material and can be obtained by the slopes of polarization, *P* and strain of *ε.* Therefore, polarization at different strains was calculated and curves of the polarization and strain were obtained for BN nanosheet, as shown in Figure 9.

From the above results, it can be deduced that the piezoelectric constant of BN nanosheet was *e* = 2.65 × 10^−10^ C/m, in the range of 1.38 × 10^−10^ − 3.71 × 10^−10^ C/m, which correlates well with the previous study [4]. The thickness of BN nanosheet was 0.33 nm during simulation and piezoelectric coefficient of the monolayer BNHCs was calculated as *e* = 0.79 C/m^2^. Some researchers have provided the piezoelectric coefficient of *e* = 1.92 × 10^−10^ C/m for a BN nanosheet by molecular dynamics simulations. Since the thickness was 0.95Å, the piezoelectric constant after removing the thickness effect was attained as *e* = 2.1 C/m^2^ [19].

**Figure 10:**
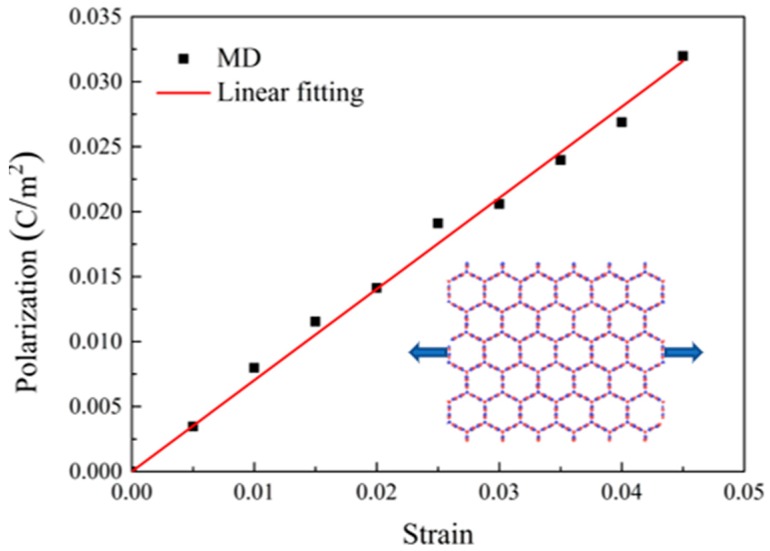
The polarization-strain curves of BNHCs under tensile loading along the zigzag direction obtained from MD simulations.

Since BNHCs is composed of BN nanosheets, polarization of BNHCs, which is due to the piezoelectric effect, can be achieved as the summation of polarization of BN nanosheets. According to the above methods, the piezoelectric properties of multilayer BNHCs were analyzed and the curves of *P* and *ε* for BNHCs were obtained, as shown in Figure 10. The piezoelectric constant of the multilayer BNHCs was calculated as $e=0.702{\;C/m}^{2}$. Findings revealed that the piezoelectric coefficient of the multilayer BNHCs was slightly smaller than that of the monolayer counterpart. The classical piezoelectric theory [30] mentions that when an external pressure is applied to a piezoelectric material, its internal structure would be deformed, resulting in separation of the positive and negative ions inside the material and formation of a dipole. The opposite magnetic poles in the material cancel each other and charges may appear on the surface of the material. According to this theory, it be concluded that polarization between the inner of BNHCs canceled each other, which may cause the polarization phenomenon of the multilayer BNHCs to be smaller than that of the monolayer counterpart, resulting in a slightly smaller piezoelectric coefficient.

## 4. Conclusions

In this paper, the mechanical properties of BNHCs under tensile loading in three different directions of zigzag, armchair and axial directions were studied using molecular dynamics simulations. Effects of temperature and strain rate on the tensile mechanical properties were analyzed. The piezoelectric properties of BNHCs in the zigzag direction were also studied. Our results showed that BNHCs possesses excellent mechanical properties along with large failure strain. The failure strains of 0.83, 0.78 and 0.55 were attained in the armchair, zigzag and axial directions, respectively. The structure also showed a high tensile strength with maximum tensile strengths of 310, 156 and 247 GPa in the zigzag, armchair and axial directions, respectively and Young’s modulus in the three mentioned directions were 146, 173 and 334 GPa, respectively. In the temperature range of 100–700 K, the mechanical properties of BNHCs at low temperature were higher than those at a high temperature. The maximum tensile strength and failure strain of BNHCs in the zigzag direction at room temperature were the highest, indicating the best strength at room temperature. In the other two directions, the maximum tensile strength and failure strain were decreased with enhancing temperature. At the initial stage of stretching, the strain rate had a little effect on the tensile properties. As the strain was increased to near the failure strain, the strain rate affected the maximum tensile strength and failure strain of BNHCs. In the armchair direction, as the strain rate was increased, the stress also inclined. Whereas, the stress was first decreased and then increased in the zigzag and cell-axis directions as the strain rate was grown up. The structure possessed an excellent piezoelectric effect due to its special internal polarization. The piezoelectric coefficient of the monolayer BNHCs was calculated as $e=0.79{\;C/m}^{2}$ and the piezoelectric coefficient of 0.702 C/m^2^ was attained for BNHCs with the thickness of 29.54 Å, which is slightly lower than that of the monolayer BNHCs.

The results of this study showed that BNHCs possessed excellent mechanical and piezoelectric properties and it may have great applications in new nanodevices with ultrahigh tensile mechanical properties and ultralight-weight materials.

## Figures and Tables

**Table 1:** Mechanical proprieties of BNHCs under the tensile loading along different directions.

Stretching Direction	Young’s Modulus (GPa)	Failure Strain	Maximum Tensile Strength (GPa)
zigzag-*x*	146	0.78	310
armchair-*y*	173	0.83	156
axial-*z*	334	0.55	247

## Supplementary Materials

The following are available online at https://www.mdpi.com/2079-4991/9/7/1044/s1, Figure S1: The curves of total energy versus temperature during heating of BNHCs; Video S1: Tensile in zigzag direction; Video S2: Tensile in armchair direction; Video S3: Tensile in cell axis direction.
