# Supplementary material for: Molecular Dynamics Simulation on Mechanical and Piezoelectric Properties of Boron Nitride Honeycomb Structures
Source: Nanomaterials (Basel). 2019 Jul 21;9(7):1044. doi: 10.3390/nano9071044 (PMC6669531; doi:10.3390/nano9071044)
Supplement: Supplementary file 1 [file nanomaterials-09-01044-s001.zip › nanomaterials-550095-suppl-final/nanomaterials-550095-supplementary-final.pdf]

Supplementary Information

Molecular Dynamics Simulation on Mechanical and Piezoelectric Properties of Boron Nitride Honeycomb Structures

Lu Xie [1,*], Tianhua Wang [1], Chenwei He [2], Zhihui Sun [1,*] and Qing Peng [3,*]

[1] School of Mechanical Engineering, University of Science and Technology Beijing, Beijing 100083, China
[2] Reactor Engineering and safety research center, China nuclear power technology research institute Co., Ltd., 518031 Shenzhen, China
[3] Nuclear Engineering and Radiological Sciences, University of Michigan, Ann Arbor, MI 48108, USA
* Correspondence: xielu@ustb.edu.cn (L.X.); sunzhihui@ustb.edu.cn (Z.S.); peng@umich.edu (Q.P.)

The Calculation of Polarization

Molecular dynamics simulations are used to calculate the poloarization of boron nitride honeycomb (BNHC) using LAMMPS (64-bit 8 Mar 2018-MPI) [1].The interactions between B and N atoms are described by Tersoff potential [2]. Time step is 0.5 fs. Periodic boundary conditions are performed in all the three directions. The sizes of simulation box are 30.32 Å × 35.01 Å × 29.52 Å containing 2016 atoms. The conjugate gradient algorithm is used to calculate the energy minimization of BNHCs to obtain the stable state of the BNHCs. The isothermal-isobaric (NPT) ensemble is utilized to control pressure (zero) and temperature (300 K) using Nose-Hoover thermostat and barostat, respectively. The relax time is 25 ps.

At a temperature of 300 K, a strain load is applied to the zigzag direction of BNHCs, and the polarization P and strain ε in the zigzag direction were recorded. The piezoelectric constant e is obtained by calculating the slopes of the polarization P and strain ε [3]. Polarization intensity can be defined as:

$$P = \sum_{i=1}^{N} x_i \cdot q_i \bigg/ V \tag{1}$$

where N is the number of atoms, and V is the volume of the model, x_i and q_i are, respectively, the displacement and the charge along the zigzag direction of atom i [4].

The calculated piezoelectric constant of the BN nanosheet is $e = 2.65\times10^{-10}$ C/m, in the range of $1.38\times10^{-10} - 3.71\times10^{-10}$ C/m, which is consistent with the previous study [5]. The thickness of the BN nanosheet is 0.33nm.The piezoelectric coefficient of the monolayer BNHCs was calculated to be $e = 0.79$ C/m^2, and the piezoelectric coefficient of the BNHCs with a thickness of 29.54 Å was calculated to be $e = 0.702$ C/m^2, which is slightly lower than that of the monolayer BNHCs.

The Comparison of Potentials

We tested three potentials of Tersoff [2], extep [6] and Reaxff [7] for boron nitride honeycomb (BNHC). The curves of the total energy and temperature of BNHCs were obtained at a heating rate of 4.7 × 10^{12} K s^{-1},as shown in Figure S1. Due to the different order of magnitude of the coordinate dimensions, in order to better show the trend of the curves, we put the curves in two different pictures.

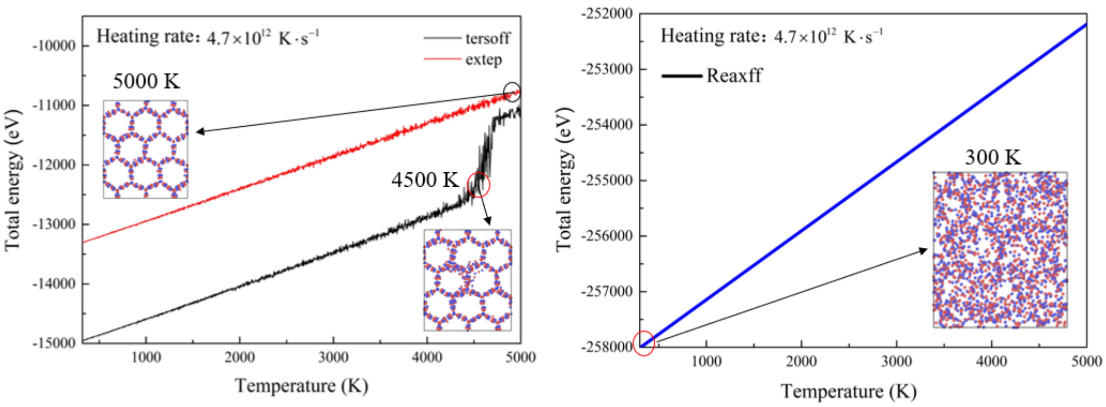

Figure S1. The curves of total energy versus temperature during heating of BNHCs.

As can be seen from Figure S1, when the Rexff potential is used to simulate the BNHC system, it cannot maintain a complete honeycomb structure at the beginning, which is not in line with the requirements of our research system. When the extep potential is used to simulate the BNHC system, it can be clearly seen that no phase change occurs, which means that when the temperature rises to 5000 K, no melting point appears. The melting point of boron nitride is about 3000 K, and the simulated value is higher than the actual melting point. However, the fact that 5000 K does not melt is not practical. Moreover, when using the Tersoff potential simulation, at 4500K, the total energy was inclined significantly and the temperature was continued to increase, the system-energy was also enhanced. This finding indicated that the phase of BNHC structure was changed at about 4500 K and it began to melt. In summary, we use the Teesoff potential to describe the interactions between B and N atoms.

Supplementary Videos

The tensile processes and the evolution of shear stains of BNHCs in the zigzag, armchair and cell axis directions are presented in Video S1, Video S2 and Video S3, respectively. The tensile strain ranges from 0 to 0.85 and BNHC fractures at 0.78 strain in the zigzag direction. The shape of the BNHC hole changes from regular hexagonal to rectangle. The tensile strain ranges from 0 to 0.85 and BNHC fractures at 0.83 strain in the armchair direction and it ranges from 0 to 0.55 in the cell axis direction. The failure strain of BNHCs under the tensile loading is 0.55 in the cell axis direction.

Reference

1. Plimpton S. Fast Parallel Algorithms for Short-Range Molecular Dynamics. Journal of Computational Physics. 1995;117(1):pp.1-19.
2. Tersoff J. Modeling solid-state chemistry: Interatomic potentials for multicomponent systems. Physical Review B. 1989;39(8):pp.5566-5568.
3. Arnau A, Soares D. Fundamentals of Piezoelectricity. Piezoelectric Transducers and Applications. 2008;pp.1-38.
4. Zhang J. Small-scale effect on the piezoelectric potential of gallium nitride nanowires. Applied Physics Letters. 2014;104(25): pp.253110.
5. Duerloo K-AN, Ong MT, Reed EJ. Intrinsic Piezoelectricity in Two-Dimensional Materials. The Journal of Physical Chemistry Letters. 2012;3(19):2871-2876.
6. Los JH, Kroes JMH, Albe K, et al. Extended Tersoff potential for boron nitride: Energetics and elastic properties of pristine and defective h-BN. Physical Review B. 2017;96(18):pp.184108.
7. Weismiller MR, van Duin ACT, Lee J, et al. ReaxFF Reactive Force Field Development and Applications for Molecular Dynamics Simulations of Ammonia Borane Dehydrogenation and Combustion. The Journal of Physical Chemistry A. 2010;114(17):pp.5485-5492.
